# Reining in Unnecessary Admission EKGs: A Successful Interdepartmental High-Value Care Initiative

**DOI:** 10.7759/cureus.18351

**Published:** 2021-09-28

**Authors:** Brendan Appold, Jennie Soniega-Sherwood, Riaad Persaud, Rachel Moss, Mityanand Ramnarine, Sean P LaVine, Rohan Bhansali, Seungjun Ahn, Mark Richman

**Affiliations:** 1 Emergency Medicine, University of Michigan School of Medicine, Ann Arbor, USA; 2 Emergency Medicine, University of California Davis School of Medicine, Sacramento, USA; 3 Emergency Medicine, Northwell Health Long Island Jewish Medical Center, New Hyde Park, USA; 4 Emergency Medicine, Citrus Memorial Hospital, Inverness, USA; 5 Internal Medicine, Northwell Health Long Island Jewish Medical Center, New Hyde Park, USA; 6 Cardiology, Northwell Health Long Island Jewish Medical Center, New Hyde Park, USA; 7 Biostatistics Unit, Feinstein Institute for Medical Research, Manhasset, USA; 8 Department of Biostatistics, University of Florida, Gainesville, USA; 9 Internal Medicine, Icahn School of Medicine at Mount Sinai, New York City, USA

**Keywords:** boarding, ekg, electrocardiogram, evidence-based medicine, guideline, educational intervention

## Abstract

Introduction

Unnecessary “admission electrocardiograms (EKGs)” on admitted patients waiting (“boarding”) in the emergency department (ED) are often ordered. We introduced evidence-based EKG ordering guidelines and determined changes in the percent of patients with "preadmission" and "admission" EKGs ordered before vs. after guideline introduction and which patient characteristics predicted EKG ordering.

Methods

In 2016, our ED, cardiology, and hospitalist services implemented EKG ordering guidelines to reduce unnecessary ED EKGs ordered after disposition. We compared pre- vs. post-guideline EKG ordering to determine whether guidelines were associated with changes in "preadmission" or "admission EKG" ordering. Patients with an admission diagnosis unrelated to cardiac or pulmonary systems were included. An EKG was “admission” if the order time was after disposition time. The numerator was the number of "admission EKGs" ordered; the denominator was the total number of such admissions; those with "preadmission EKGs" were excluded from this analysis. Variables that might influence EKG ordering were explored. The chi-square test with Bonferroni adjustment was used to compare 2015 vs. 2016 percentages of patients with an “admission EKG.”

Results

There was a decrease in unwarranted "admission EKGs" among ED boarding patients (44.1% pre-implementation to 27.5% by two years post-implementation) and an increase in unwarranted "preadmission EKGs" (66.1% pre-implementation to 72.8% post-implementation). Age ≥40 and past medical history independently predicted EKG ordering.

Discussion

The decrease in the ordering of "admission EKGs" but "preadmission EKGs" suggests the decline reflects a true change in ordering and not a general environmental/ecologic decline in ordering. This highlights the importance of careful guideline development and implementation.

## Introduction

Admitted patients often wait (“board”) for many hours in the emergency department (ED) for an inpatient bed after the decision to admit to the hospital is made, contributing to morbidity, mortality, and patient, provider, and staff dissatisfaction [1]. Such patients often undergo “admission” studies, ordered and performed in the ED after the decision to admit is made but without clinical prompting on the basis of history, physical examination, or results from prior objective studies. These studies are often ordered according to hospital policy or on the admitting provider’s belief. Each admitted patient needs a common set of routine admission studies (e.g., complete blood count, chemistry panel [2], and chest X-ray (CXR)) [3]. Likewise, “admission electrocardiograms (EKGs)” are routinely ordered on patients without indications. In one study of 636 admissions from the ED, 202 had EKGs performed without indication; EKG changed management in only three (1.5%) and affected outcomes in none [4]. An admission EKG differs from a pre-admission EKG ordered in the ED on the basis of clinical suspicion and from a "post-admission EKG" ordered once the patient is in an inpatient unit.

EDs often get requests from admitting services for further testing and consultations prior to admission, adding to delays in patients departing the ED [5]. Evidence-based guidelines from the American College of Cardiology (ACC)/American Heart Association (AHA) [6], the U.S. Preventive Services Task Force (USPSTF) [7], and the American Academy of Family Physicians (AAFP) [8] do not encourage EKGs for asymptomatic patients at low risk for coronary artery disease events.

The downstream consequences of clinically unsuspected abnormal EKGs, and a misdiagnosis of electrocardiographic artifacts, are substantial and can lead to inconveniences, costs, and possible risks to patients, including specialty consultation, coronary calcium CT, echocardiogram, cardiac stress test, and coronary artery catheterization [9]. Cardiac catheterization has a complication rate of 1%, including vascular access hematoma, pseudoaneurysm, arteriovenous fistula, coronary artery dissection, and thrombosis and embolism [10]. A recent article of surveyed physicians found cascades of care after incidental findings to be common (90% of respondents) and caused physical harm (15.6%), a financial burden (57.5%), wasted time and effort (69.1%), and caused frustration (52.5%) and anxiety (45.4%). One-third stated that the test revealing their most recent incidental finding that triggered a cascade may not have been clinically appropriate. Guidelines for follow-up testing were not followed (8.1%) or did not exist, to their knowledge (53.2%). Nearly two-thirds of respondents chose accessible guidelines and 50% chose decision aids as potential solutions [11].

These potential downfalls were confirmed in a recent large, population-based cohort study that found low-risk patients who had an EKG were five times more likely to receive a specialty consultation or cardiac test or procedure than those who did not, suggesting extraneous EKGs can trigger a diagnostic cascade where higher rates of non-invasive diagnostic testing lead to higher rates of more-invasive diagnostic testing and therapeutic interventions [12].

False-positive studies (e.g., screening mammography) are associated with unnecessary patient anxiety [13], financial output and distress [14], and delays in subsequent care [15]. Targeting testing to more appropriate groups can reduce false-positive rates, and thereby enhance patients’ experience with the healthcare system.

The negative implications of unwarranted EKGs are particularly problematic given that they provide little clinical value. The same study found cardiac event rates of less than 1% for both members of the group that received EKG testing and those who did not. The typical cost for performing and interpreting an EKG is $100; given the generally brief time it takes to perform and interpret an EKG, this test is relatively expensive and the frequency of these common tests leads to accumulated costs. Only 1% of EKGs on patients without a clinically evident cardiac abnormality provide useful information, costing $6,700 per EKG abnormality, with only approximately one in 400 patients (0.26%) receiving lasting benefit from the EKG [16].

In an era where rising healthcare costs have led to an increased focus on value-based care, acting on evidence-based recommendations for EKG utilization provides an opportunity for cost savings and improved patient experience, with minimal drawbacks.

In our experience, practice styles at our particular hospital follow long-standing cultural norms, despite updates in evidence (eg, admission chest X-rays continued to be ordered commonly on patients without cardiopulmonary complaints, despite little evidence of value [17]; creatine kinase (CK) and CK-MB were routinely ordered in diagnosing acute coronary syndrome, despite troponin being adequate in almost all cases [18]. Thus, culture, or tradition, was the most likely foundational factor explaining why unnecessary admission EKGs continued to be routinely ordered. The authors sought to address this through cultural change driven by a locally developed and approved guideline, which was the most appropriate intervention.

In 2016, the ED, cardiology, and internal medicine hospitalist services of the Northwell Health Long Island Jewish Medical Center (LIJMC) implemented guidelines (Appendix) to reduce EKGs ordered in the ED after admission decision if the chief complaint, history/physical exam, and diagnosis did not suggest an EKG was warranted. The intended effect of the guidelines was to remove the concept and performance of admission EKGs performed routinely when a patient is admitted to the hospital, not prompted by clinical suspicion for cardiac disease. After the introduction of the guidelines, the concept of admission EKG was removed and replaced with appropriate indications for an EKG to be performed in the ED during or following the ED evaluation. A preadmission EKG remained "an EKG performed, as needed, during the ED visit or consultation on the basis of clinical suspicion."

All ED and hospitalist providers were educated on the guideline. We studied the impact on future admission EKG ordering for such patients.

The primary aim of this study was to determine if there were changes in the percent of patients on whom preadmission and admission EKGs were ordered following the introduction of the guidelines. Our secondary aim was to determine whether certain patient characteristics (demographics, vital signs, ED diagnoses, past medical history) might predict getting a preadmission vs. admission EKG and whether these differed between pre- and post-intervention. We investigated changes in preadmission EKG rates to determine whether any changes in admission EKG rates reflected a possible influence of ecologic changes in EKG ordering (i.e., there may have been a general change in EKG ordering rates) rather than the specific impact of this intervention to decrease unnecessary admission EKG ordering.

## Materials and methods

LIJMC is a 500-bed tertiary-care academic hospital, serving a racially and socioeconomically diverse population, with a large proportion of geriatric patients. Two attending physicians from the ED, one from cardiology, and one from the internal medicine hospitalist service reviewed the literature regarding admission EKGs. In June 2016, the departments implemented joint guidelines (Appendix) to reduce extraneous EKGs ordered in the ED after admission decision if the chief complaint, history/physical examination, and diagnosis did not suggest an EKG was warranted. All ED and hospitalist attending, resident, and mid-level providers were educated once about the guideline via announcement at the ED morning briefing and the hospitalist monthly meeting. We evaluated the translation of the guideline into practice by investigating the impact of the new guideline and education initiative on future extraneous admission EKG ordering.

We performed a retrospective analysis of data from July-December 2015 (pre-guideline) vs. July-December 2016 and July-December 2018 (post-guideline). Patients were included if admitted during those timeframes with an admission diagnosis unrelated to the cardiac or pulmonary systems: adverse drug effect, allergy, anemia, back pain, infections (eg, cellulitis, enteritis, meningitis, pyelonephritis), dehydration, diabetes, migraine, multiple sclerosis, nausea/vomiting, and pancreatitis. An EKG was considered an admission EKG if the order time was after the electronic health record (EHR) admission disposition time. The numerator was the number of admission EKGs ordered on patients with diagnoses unrelated to the cardiac or pulmonary systems (subtracted by those with a preadmission EKG); the denominator was the number of such admissions (subtracted by those with a preadmission EKG).

Variables of interest that might influence whether an EKG was ordered were: age, gender, vital sign abnormalities, chief complaint, ED diagnosis category, or relevant past medical history. Based on experience, the two ED investigators decided vital sign abnormalities that might prompt an EKG were: systolic blood pressure >180 mmHg, diastolic blood pressure >105 mmHg, heart rate >110 beats/minute, and respiratory rate >24/minute. ED diagnoses were categorized as follows: allergy; back pain; dehydration; electrolyte abnormality, metabolic derangement, hyperglycemia; gastrointestinal; infectious; and neurologic. We searched for the following keywords in the patients’ past medical history that might prompt an EKG, even in the absence of current cardio-pulmonary complaints: heart disease (coronary artery disease, myocardial infarction, atrial fibrillation, congestive heart failure), renal disease (renal failure, acute kidney injury, end-stage renal disease, dialysis, hyperkalemia), or neurovascular conditions (transient ischemic attack, stroke, cerebrovascular accident). We also investigated whether, among patients with preadmission EKGs, there were differences pre- vs. post-guideline implementation in the percent of patients with a chief complaint that might have triggered a preadmission EKG.

The chi-square test was used to compare 2015 vs. 2016 percentages of patients with an admission EKG. Upon finding a significant difference (p<0.05), the Bonferroni adjusted pairwise chi-square test was utilized to compare 2015 vs. 2016 percentages having a preadmission vs. an admission EKG.

A comparison of EKG status (preadmission vs. admission) for each separate predictor variable was performed using univariable multinomial logistic regression for each year separately. Predictor variables that were significantly associated with an EKG being ordered were entered into the multivariable model. Tukey adjusted pairwise multiple comparisons were performed for the diagnosis categories variable. Results were considered statistically significant a priori at p<0.05. Analysis was conducted using SAS v. 9.4 (SAS Institute, Inc., Cary, NC). This study was deemed exempt from IRB review and approval (Northwell Health Human Research Protection Program’s Human Subjects Research Determination Request (HSRD HSRD20-0341).

## Results

Between July and December 2015, there were 1,174 admissions with relevant diagnoses; between July and December 2016, there were 577; and between July and December 2018, there were 454. The large decrease in admissions was a result of the use of decision-support tools (eg, CURB-65) [19] and new hospital policies encouraging the use of a short-stay, ED-run observation unit for patients whose expected length of stay would be under 24 hours. Pre- and post-guideline (i.e., 2015 vs. 2016) patients had similar gender distributions, as well as clinical characteristics (abnormal vital signs; past medical history (PMH) of conditions for which ED EKGs are routinely ordered (e.g., cardiac, chronic kidney disease), with the exception of a slight increase in the percent of patients ≥40 years old (Table 1).

**Table 1:** Patient demographic and clinical characteristics, pre-guideline (2015) vs. post-guideline (2016)

Characteristic	Pre-guideline (2015)	Post-guideline (2016)	P-value
Age (≥40 years)	88.80%	92.30%	0.018
Gender	46.80%	48.40%	0.53
Vital signs	23.50%	24.30%	0.73
PMH	41.30%	42.60%	0.6

**Figure 1:**
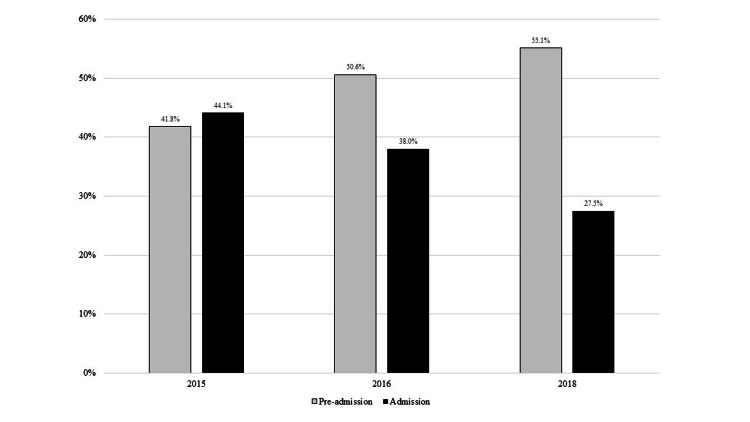
Percent of patients with preadmission and admission EKG, pre (2015) vs. post (2016, 2018)-guideline

**Figure 2:**
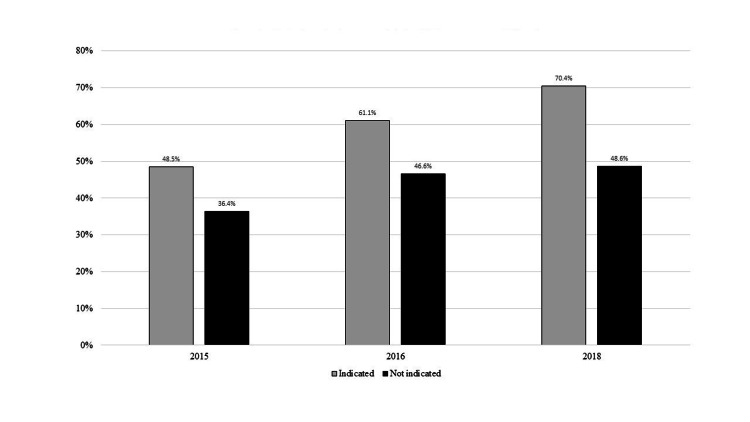
Percent of patients in whom a preadmission EKG was, or was not, indicated

Among patients seen in 2015, 44.1% had an admission EKG performed in the ED; this decreased in 2016 to 38.0% (p=0.0151, 6.1% absolute decrease, 13.8% relative decrease) and to 27.5% in 2018 (p<0.0001 for 2015 vs. 2018, 16.6% absolute decrease, 37.6% relative decrease). In contrast, the percent of patients with preadmission EKGs increased from 41.8% in 2015 to 50.6% in 2016 (p=0.0005) and to 55.1% in 2018 (p<0.0001 for 2015 vs. 2018) (Figure 1). The change in preadmission EKGs was significantly different from the change in admission EKGs between 2015 and 2016 (p<0.0018). The percent of patients with a chief complaint suggesting a preadmission EKG was indicated and who actually had a preadmission EKG increased from 48.5% in 2015 to 61.1% in 2016 (p=0.0075) and 70.4% in 2018 (p<0.0001 for 2015 vs. 2018). However, the percent of patients without a chief complaint indicating an EKG might be appropriate and who actually had a preadmission EKG ordered also increased, from 38.4% in 2015 to 46.6% in 2016 (p=0.006) and to 48.6% in 2018 (p=0.0019 for 2015 vs. 2018) (Figure 2). Age ≥40, abnormal triage vital signs, and past medical history of cardiovascular or renal disease (unrelated to the reason for admission) were associated with an increased likelihood of a preadmission or admission EKG in both 2015 and 2016 (Table 2).

**Table 2:** Odds ratios of demographic and clinical characteristic influence on the likelihood of preadmission and admission EKGs in the pre-guideline (2015) and post-guideline (2016) time periods

Characteristic	Pre-guideline (2015) “pre-admission” EKG	Post-guideline (2016) “pre-admission” EKG	Pre-guideline (2015) “admission” EKG	Post-guideline (2016) “admission” EKG
Age (≥40 years)	5.2 (3.2 – 8.6)	2.9 (1.3 – 6.6)	3.0 (1.9 - 4.7)	2.1 (0.9 – 4.9)
Gender	0.78 (0.54 – 1.1)	1.2 (0.7 – 2.1)	0.83 (0.58 – 1.2)	1.0 (0.60 – 1.8)
Vital signs	1.7 (1.1 – 2.7)	2.1 (1.0 – 4.3)	1.6 (1.0 – 2.6)	1.7 (0.8 – 3.5)
PMH	26.4 (11.5 – 60.8)	20.3 (8.7 – 47.2)	21.8 (9.5 – 50.2)	17.6 (7.6 – 40.7)

Change in length of stay (LOS) pre- vs. post-guideline implementation

Between 2015 and 2018, among patients with a preadmission EKG (but no admission EKG), the median total ED LOS decreased from 11.6 to 8.7 hours (p<0.0001); the median boarding ED LOS decreased from 5.2 to 3.6 hours (p=0.0001). Among patients with a postadmission EKG, the median total ED LOS decreased from 11.8 to 8.6 hours (p<0.0001); median boarding ED LOS decreased from 5.6 to 3.5 hours (p<0.0001). These changes in LOS were seen only between 2015 vs. 2018, but not between 2015 vs. 2016, for both preadmission and admission EKGs.

Impact on cost

After adjusting for the decrease in eligible patients, the reduction in admission (boarding) EKGs was associated with a cost savings of $7,161.40 in 2016 and $12,327 in 2018 compared to what would have been spent had 2015 admission EKG rates been maintained (rather than reduced) in 2016 and 2018.

## Discussion

This educational initiative to improve appropriate EKG ordering was associated with a decrease in admission EKGs among ED boarding patients without a pre-admission EKG and whose admission diagnosis did not suggest an EKG was warranted (44.1% pre-implementation to 27.5% by two years post-implementation). During the same time, there was an increase in preadmission EKGs among patients whose chief complaint and admission diagnosis did not suggest one was warranted (from 66.1% pre-implementation to 72.8% post-implementation). The decline in admission EKGs is not likely due to the increase in preadmission EKGs, as these are two separate populations (patients with a preadmission EKG were excluded from the denominator of the admission EKG analysis). Rather, the decline in admission EKGs is likely due to a true change in ordering behavior. There was an increase in preadmission EKGs ordered without clinical indication (38.4% in 2015 vs. 46.6% in 2016 (p=0.006) vs. 48.6% in 2018 (p=0.0019 for 2015 vs. 2018). Pre- vs. post-implementation populations (Table 1) were similar. The decrease in admission, but not preadmission, EKG ordering suggests the decline was due to the targeted nature of this intervention and not to a general environmental/ecologic decline in EKG ordering nor a change in population or clinical characteristics.

Patients with a past medical history of cardiovascular, renal disease, or hyperkalemia whose admission diagnoses did not suggest an EKG was necessary, and who already had a thorough history and physical examination by a physician, were 17 times as likely to receive an admission EKG than those without such PMH. It is possible the admitting physician uncovered something during their history or physical examination that warranted an EKG. It is also possible a "boarding" patient had a sudden change in status for which an EKG was appropriate; this would have been captured as an inappropriate admission EKG, even though the EKG was indicated. Our study was not designed to discover such occurrences.

At the same time, there was an increase in unwarranted preadmission EKGs. Many forces may have contributed to this increase. In our ED, many EKGs are performed by technicians prior to a provider seeing the patient, triggered by particular chief complaints (eg, chest pain, shortness of breath, altered mental status, weakness, atrial fibrillation). However, numerous preadmission EKGs were performed on patients with chief complaints that did not suggest one was warranted (e.g., cough, blurry vision, diarrhea, rash, vaginal bleeding, etc.). The Centers for Medicare and Medicaid Services (CMS) metric door-to-EKG standard for patients presenting with chest pain or (chest pain equivalent) is 10 minutes, to optimize ST-elevation myocardial infarction (STEMI) management [20]. This may lead to a “shoot first, ask questions later” approach to performing EKGs. An increasingly elderly population with complex medical problems and unclear presenting symptoms (or symptoms potentially attributable to cardiac causes) may also contribute to the increasing number of EKGs performed upon ED arrival [21].

One reason evidence-based guidelines are occasionally not followed is medico-legal concerns. Evidence-based, guideline-backed, cost-effective diagnostic guidelines reflect a reasonable balance between detecting disease in a population (true positives) vs. generating false positives. Guidelines recognize there will be some amount of risk on the part of the patient and provider. Persons below the USPSTF’s recommended ages for breast and colon cancer occasionally get cancer, for example. Guidelines regarding appropriate EKG ordering may lead to missed, incidental significant heart disease. It is reassuring, however, that prior studies found no or very few significant adverse outcomes [22-24]. In Moorman’s study of 775 screening EKGs, only two conferred lasting benefit: in one patient, the EKG led to a finding of well-tolerated pulmonary emboli; in the other patient, the EKG identified an acute myocardial infarction, the finding of which, the authors estimate, added three weeks to the patient’s life expectancy [16]. An investigation of EKGs performed at a health center found only a small percent led to changes in patient management, even in cases the EKG read “myocardial infarction,” in part because EKG readings differed from the initial consideration that prompted the EKG (i.e., there was discordance between the clinical indication for the EKG and the EKG read-out) [21]. A study of visits to a general medicine clinic with a cardiology bias found the patient’s history to be the most useful element in obtaining an accurate diagnosis, particularly for cardiac symptoms. EKGs were helpful in 66% of cardiac cases where a cardiac condition was suspected based on symptoms/signs but in only 16% of cases where no cardiac diagnosis was suspected. Even in patients with chest pain, the history decided 90% of diagnoses. The author concluded that objective studies (such as an EKG) should be obtained to answer specific clinical questions that cannot be answered by symptoms/signs alone [22].

The success of this educational intervention is likely due to several features. First, all relevant specialties were involved: emergency medicine, cardiology, and hospitalist medicine. Second, none of the three national representative organizations (representing primary care, cardiology, and the USPSTF) advocates for “screening” EKGs for asymptomatic patients (i.e., there was no conflict between organizations). Finally, in contrast to many guidelines, which encourage positive actions, this guideline encouraged doing less (i.e., not to do an EKG) for a substantial portion of patients. It is easier to adapt to recommendations not to do something than to recommendations to take action, as taking action requires remembering to act and finding time to do so with attention to detail.

Our data revealed greater improvements in median boarding and total LOS after three years than would be expected solely by reducing the percentage of patients with preadmission or admission EKGs, as EKG performance, interpretation, and documentation take only a few minutes. The disproportionate declines in LOS likely reflect other improvements in patient flow, such as a hospital-wide initiative to increase discharges before noon.

Barriers

The awareness-to-adherence model describes four stages to achieving guideline-concordant care: an awareness that a guideline exists, agreement with it, adopting the guideline, and adhering to it [25]. Previous studies have shown that, in many cases, only about half of the physicians are aware that guidelines exist (range 1% to 84%, median 54.5%). For any particular guideline, about 10% (range 1% to 91%) of physicians won’t agree with it. Adherence is influenced by the ease of use, perceived applicability to one’s patient population, degree of appreciation for physician autonomy, culture/common practice in a particular environment, point-of-care reminders, and assessment of the legal risk of adhering to the guideline [26]. In addition, the fear of legal consequences of missing significant diagnoses represents another reason why up to 90% of physicians at least occasionally order studies indications (defensive medicine) [27]. This multifactorial nature behind guideline nonadherence contributes to the frequently reported time lag averaging 17 years for research evidence to reach clinical practice [23].

This initiative might have been even more effective had we employed additional or alternate approaches to guideline development and promotion. The EKG guidelines were developed by attending physicians, whereas orders for EKGs are mostly placed by house staff. Incorporating house staff in the guideline development process may have made implementation more effective. Under attending supervision, house staff could have performed the literature search and drafted the guidelines. A study published in the Journal of General Internal Medicine involving 79 physicians in an acute-care teaching hospital evaluated the effects of guidelines developed by members of the affected group and agreed upon by consensus, to standardize the treatment of 14 diagnoses. Data analysis revealed a significant decrease in EKG ordering, attributable to the practice guideline, which was sustained six months following implementation [28]. Improvements in compliance of up to 40% have been seen when guidelines are developed by consensus, by end-users, or by persons with high perceived credibility [29]. Guideline development by end-users or consensus allows physicians to feel ownership of the recommendation and can facilitate clinician adherence despite conflicting or weak recommendations of professional bodies.

A multimodal education program, rather than a single strategy, might have proven more effective than solely announcements. Many researchers reported greater efficacy of multifaceted vs. single-strategy implementation [28]. Announcements are a traditional passive education and information dissemination strategy and are usually ineffective in guideline implementation. Interactive educational strategies, such as meetings and workshops, are associated with successful implementation. House staff could have presented the guidelines to colleagues at educational conferences, using case studies, asking the audience if they would order an EKG for each case, and discussing in which circumstances ordering an EKG is appropriate.

In addition, in our intervention, education about proper EKG ordering was provided only once via announcements at the ED morning briefing and hospitalist monthly meeting. However, house staff turn over every year, as some graduate residency and recent medical school graduates begin internship. This project may have been more successful had the educational campaign been repeated during the same academic year, and again at the start of each new academic year. Strategies that incorporate subsequent reminders and follow-up education, as well as audit or peer review, are particularly successful in decreasing the number of diagnostic tests performed [28]. The benefit of reminder education was demonstrated in an initiative to decrease CT scans ordered for patients with minor head injuries, which was successful in reducing imaging rates in the same EDs when implemented with an hour-long education session and reminders [30].

Involving service-line thought leaders in the reminder process would likely make them even more effective. Effective leadership, in addition to repeated educational interventions and reminders, is important to guideline implementation [31]. Several studies have identified a diffusion process in which clinical opinion leaders can promote behavioral change through social influence in their local networks. When guidelines are implemented, practitioners often seek information and opinions from their peers rather than individually reviewing the data driving a recommendation. Clinical opinion leaders can lend expertise in identifying barriers to change and assist in implementation strategies [28,32]. In this initiative, follow-up reminders via email or online education activities could have been created by the LIJMC ED, hospitalist, and cardiology thought leaders.

Finally, a guideline cannot replace clinical decision-making or cover every scenario a clinician might encounter. Consider a patient sent to the ED from their primary care physician with an abnormal EKG that is then misplaced; an EKG may be requested by the admitting team after the decision-to-admit time and therefore appear to be an admission EKG. We were not able to account for such events in our analysis.

Limitations

We did not interview (individually or via focus groups) ordering providers to investigate why admission EKGs were being ordered. However, from the authors’ informal discussion with ED and admitting providers, culture, or tradition, was most commonly mentioned as the foundational reason for the routine ordering of unnecessary admission EKGs. Additionally, we did not review the entire chart for preadmission EKG indications other than the chief complaint, initial vital signs, PMH, and ED diagnosis. It is possible that a review of the documented history, review of systems, and physical examination may have uncovered indications such as chest pain or shortness of breath. In addition, we could not ascertain what circumstance or which service (e.g., emergency medicine vs. internal medicine) prompted the admission EKGs; it is possible a more thorough history and physical examination performed by the admitting internal medicine team revealed a previously undiscovered appropriate indication for an EKG.

## Conclusions

Although guidelines advise against routine EKGs for low-risk, asymptomatic patients, they continue to be ordered by many clinicians as standard practice. Our interdepartmental collaboration was associated with a decrease in ordering extraneous admission EKGs among ED boarding patients, though there was an increase in preadmission EKGs. This study highlights the importance of the careful development and implementation of clinical guidelines.

## Appendices

Guideline for appropriate ordering of emergency department EKGs 

Guideline

1. Patients with a cardiopulmonary complaint (or a complaint often related to a cardio-pulmonary condition (e.g., weakness, dizziness, abdominal pain in elderly or diabetic patients)) should have an EKG as part of the admission evaluation.

2. Patients expected to have procedures in which a pre-procedure cardiac evaluation is standard should have an EKG as part of the admission evaluation.

3. Patients without a cardio-pulmonary complaint (or a complaint often related to a cardio-pulmonary condition (e.g., weakness, dizziness, abdominal pain in elderly or diabetic patients)), and who are not expected to have procedures in which a pre-procedure cardiac evaluation is standard, do not require an EKG upon admission.

4. EKGs on patients without a cardio-pulmonary complaint (or a complaint often related to a cardio-pulmonary condition (eg, weakness, dizziness, abdominal pain in elderly or diabetic patients)), and who are not expected to have procedures in which a pre-procedure cardiac evaluation is standard, can be requested or performed on a case-by-case basis at the provider's discretion; a patient can be assigned a level of inpatient care, called up to, and accepted by a medical team prior to the EKG being performed.

Background

Occasionally, admitting services ask for an admission EKG in patients whose history and physical examination do not suggest a cardiac abnormality. Common reasons for requesting an admission EKG include detecting underlying cardiac disease that could explain the patient's symptoms and establishing a baseline EKG for reference in the event the patient has a cardiac complaint during the hospital course. We investigated the literature regarding baseline EKGs for hospitalized patients to determine whether there is a sub-population for whom admission EKGs should be performed in patients whose history and physical examination do not suggest a cardiac abnormality or whether the practice of admission EKGs should be abandoned.

Evidence

1. Individual Articles

a. Moorman JR, Hlatky MA, Eddy DM, Wagner GS. The yield of the routine admission electrocardiogram. A study in a general medical service. Ann Intern Med. 1985;103(4):590-5 [16]. In this study, 1,410 patients admitted to a general medical service were studied. In 775 patients with no evidence of cardiac abnormality, 8 screening EKGs (1%) added information. Among patients whose history and physical examination did not suggest a cardiac abnormality, the EKG yield (added information) was 1% and cost-effectiveness (1985 dollars) was $24,000 per year of life saved ($53,760 in 2016 dollars). Only age and clinical suspicion for cardiac disease predicted the yield of the EKG. The EKG among older patients (≥ 45) was more helpful than in patients <45, but this finding was of only borderline statistical significance (0.05 < p < 0.10) when adjusted for the presence of a cardiac abnormality (ie, if the patient didn’t have a cardiac abnormality, there was no statistical evidence of yield for the EKG).

b. Rubenstein LZ, Greenfield S. The baseline ECG in the evaluation of acute cardiac complaints. JAMA. 1980; 244(22):2536-9 [33]. In this study, 236 ED patients were evaluated to determine whether having a baseline EKG would have affected disposition. There was no patient for whom a baseline EKG would have aided in avoiding an inappropriate discharge. That is, a prior EKG would not have affected management in a more conservative direction to avoid an inappropriate discharge. For admitted patients, a baseline EKG would clearly not affect disposition decisions. Should the patient develop cardiac symptoms as an inpatient, most likely an EKG and, perhaps, cardiac enzymes, would be ordered; a baseline EKG would not encourage the provider to be more conservative and not order these studies.

2. Position Statements by Organizations

a. American Academy of Family Physicians Summary of Recommendations for Clinical Preventive Services. July 2017. https://www.aafp.org/dam/AAFP/documents/patient_care/clinical_recommendations/cps-recommendations.pdf [8]. The AAFP recommends against screening with resting or exercise electrocardiography (ECG) for the prediction of coronary heart disease (CHD) events in asymptomatic adults at low risk for CHD events. (2012) (Grade: D recommendation). The AAFP concludes that the current evidence is insufficient to assess the balance of benefits and harms of screening with resting or exercise ECG for the prediction of CHD events in asymptomatic adults at intermediate or high risk for CHD events. (2012) (Grade: I recommendation).

b. Greenland P, Alpert JS, Beller GA, et al.; American College of Cardiology Foundation/American Heart Association Task Force on Practice Guidelines. 2010 ACCF/AHA guideline for assessment of cardiovascular risk in asymptomatic adults: executive summary: a report of the American College of Cardiology Foundation/American Heart Association Task Force on Practice Guidelines. Circulation. 2010; 122(25):2748-64 [6]. A resting electrocardiogram (ECG) is reasonable for cardiovascular risk assessment in asymptomatic adults with hypertension or diabetes. (Level of Evidence: C). A resting ECG may be considered for cardiovascular risk assessment in asymptomatic adults without hypertension or diabetes. (Level of Evidence: C).

c. Moyer VA. U.S. Preventive Services Task Force. Screening for coronary heart disease with electrocardiography: U.S. Preventive Services Task Force recommendation statement. Ann Intern Med. 2012; 157(7):512-8 [7]. The USPSTF recommends against screening with resting or exercise ECG for the prediction of CHD events in asymptomatic adults at low risk for CHD events (D recommendation). The USPSTF concludes that the current evidence is insufficient to assess the balance of benefits and harms of screening with resting or exercise ECG for the prediction of CHD events in asymptomatic adults at intermediate or high risk for CHD events (I statement).
